# Chicago Medical Society

**Published:** 1883-09

**Authors:** 


### ARTICLE VIII.

### CHICAGO MEDICAL SOCIETY.

The Chicago Medical Society held a regular meeting July 2, 1883, with the President, Dr. D. W. Graham, in the chair. The minutes of the previous meeting were approved, as read by the Secretary.

Drs. E. J. Doering and Wm. J. Gates were elected to active membership.

After additional routine business had been transacted, Dr. Simon Strausser read a paper on "How Doctors and Medical Societies Should be Made More Efficient."

#### ABSTRACT OF THE PAPER.

The life of a physician is one of great care and responsibility, more thorns than flowers are strewn in the path of the arduous duties of his profession, and he loses at least one half of his earnings by being a poor financier, or through unscrupulous persons, or by his own charitable nature, or from an ever exacting public, who frequently fail to pay him for services rendered, and then add insult to injury by defaming the character and skill of their former attending physician.

The druggist frequently uses his power to injure the independant and honest practitioner—besides depriving the doctor of a fee by taking upon himself the duty of prescribing his own medicines, or unnecessary delay in preparing what was previously prescribed by a physician, which may cause great injury to a patient or jeopardize life. A satirical remark by an elderly physician is frequently made in public of a younger brother, especially if the younger Esculapius is poor in pocket, and has just entered upon his professional career.

These are a few of the difficulties to be overcome, that beset the practitioner in his pathway, and how can this be remedied? Can the combined influence of this honorable association do anything to bring about this desired change?

Again. it is humiliating to every honest, upright, and honorable physician, to see men in our profession using political tactics for office or position, or through chicanery be called before the courts in the capacity of an expert, instead of their merits or professional ability being known, and resulting in their being called to give expert testimony. This society has frequently been held up to the public gaze in scorn for the ignorance displayed by the so called expert, thus throwing a cloud upon the fair name of our honorable calling.

We have in our midst men learned in the sciences. who are earnest and honest laborers for the advancement of professional learning, and are thoughtfully meditating how best to eradicate these evils.

Regarding our County Hospital management, it is to be regretted that it is done in the interest of political aggrandizement, the warden being appointed regardless of his fitness and ability, and this Society should use every honorable means to call public attention to this abuse. Many young lives might be saved if there were special wards provided in our hospitals for certain specific ailments.

Our united efforts should be exercised in sustaining the local and State Boards of Health, and abbatoirs and other dealers in cattle, poultry and milk should be watched with a jealous eye, thus lessening the cause of mortality. To be sure, a law exists for the punishment of those adulterating food, but is it ever enforced?

It becomes our duty to urge upon the authorities the necessity of the faithful enforcement of this law. This Society should exert its influence with the public in the erection and maintenance of a hospital for children, and to correct that greatest of all evils existing in our large cities, that causes such frightful loss of life and untold misery and disease, viz. prostitution. The writer concluded by admonishing his brethren to be true to each other, moral and conscientious, and that we unite in the advocacy of a "State Board of Examiners," with a graded and thorough examination for the degree to which we aspire, and thus we will be more efficient, individually and collectively.

In the discussion, Dr. C. T Fenn first remarked, by stating he scarcely knew which to consider in the paper, so he asked the author if the field gone over was not too extensive for the announcement, and that it was too sweeping in character; was the paper read to benefit the Society?

Dr. G. C. Paoli thought if a "Bismarck" was in this country, the writer's ideas might then be carried out. Regarding the County Hospital management, all we could do would be to agitate the question alluded to in the paper, and instruct the county commissioners as to their duties. He knew Dr. Strausser meant well by advocating what he did, but thought he was taking too dark a view of his subject.

Dr. Strausser answered both gentlemen who had precede him, and said his object in reading the paper was to benefit the Chicago Medical Society and improve us. To do so, he thought the constitution and by-laws would require first to be changed. He had the good of the profession at heart. and meant well, indeed, and offered a motion that a committee of five (5) be appointed by the chair to revise or amend the constitution and by-laws, and report at the first meeting of the Society in the fall.

The motion was seconded by Dr. A. R. Reynolds.

Chairman Graham retired temporarily, and called Dr. Paoli to the chair.

Dr. G., who was opposed to the motion, spoke in a pleasant and entertaining manner for some minutes. He did not think we ought to revise the constitution and by-laws. He thought Dr. Strausser had a dark view of everything in the Society, the same as frequently occurred to young members.

The motion was then put and carried, resulting in the appointment of Dr. S. Strausser, Dr. G. C. Paoli, Dr. C. T. Fenn, Dr. C. W. Purdy, Dr. D. R. Brower.

Miscellaneous business—none.

Adjourned. L. H. M.

